# Clinical and Pathologic Characteristics of Pulmonary Carcinoid Tumors in Central and Peripheral Locations

**DOI:** 10.1007/s12022-018-9530-y

**Published:** 2018-05-16

**Authors:** George Papaxoinis, Angela Lamarca, Anne Marie Quinn, Wasat Mansoor, Daisuke Nonaka

**Affiliations:** 1 Department of Medical Oncology, The Christie NHS Foundation Trust, Manchester, UK; 2 Department of Histopathology, Wythenshawe Hospital, Manchester NHS Foundation Trust, Manchester, UK; 3 Department of Cellular Pathology, Guy’s and St Thomas’ NHS Foundation Trust, London, UK

**Keywords:** Carcinoid tumor, Endobronchial, Sustentacular cell, OTP, TTF-1

## Abstract

Pulmonary carcinoid tumors occur in both central and peripheral locations, and some differences in clinico-pathological features have long been observed. We investigated a large number of resected carcinoid tumors with the aim to further define the characteristics of tumors from both locations. One hundred sixty-six resected carcinoid tumors of the lung were analyzed for a variety of clinical and pathologic features, including histology subtype, mitotic rate, Ki67 index, necrosis, invasive pattern, architectural pattern, cell morphology, sustentacular cells, neuroendocrine hyperplasia, and orthopedia homeobox protein (OTP) and TTF-1 immunohistochemical expressions. Unsupervised hierarchical cluster analysis suggested three clusters as the best solution using TTF-1 and OTP expression: TTF-1-positive and OTP-positive tumors as cluster 1, TTF-1-positive but OTP-negative as cluster 2, and TTF-1-negative and OTP-negative as cluster 3. Cluster 1 was characterized by peripheral location, presence of spindle cell component, presence of sustentacular cells, female predominance, and strong association with neuroendocrine hyperplasia. Cluster 2 was characterized by central location, polygonal cell morphology, acinar growth pattern in a subset of tumors, and only rare association with neuroendocrine hyperplasia. Cluster 3 consisted of more aggressive tumors with more heterogeneous pathologic features. Tumors showed polygonal cell morphology and acinar growth pattern. Occurrence of neuroendocrine hyperplasia was exceptional. Our study confirmed distinct characteristics of central and peripheral type carcinoid. An important difference was a strong association of the peripheral tumor with neuroendocrine hyperplasia while such an association in central tumors was negligible. The tumor location might be relevant for pathobiology of lung carcinoid tumors.

## Introduction

Lung carcinoid tumor belongs to a neuroendocrine tumor group and is subdivided to typical carcinoid (low grade) and atypical carcinoid (intermediate grade), based on mitotic rate and presence or absence of necrosis. A number of studies have shown this subgrouping correlates with prognosis [1]. A variety of prognostic factors and biomarkers have also been reported in the literature, including Ki67 index [2], stage [3], deletion of 11q22.3-q25 [4], CD44 [5, 6], and orthopedia homeobox protein (OTP) [5]. The carcinoid (neuroendocrine tumor/NET) of gastrointestinal tract has demonstrated significant biologic differences depending on their anatomic location within the system [7], and given the variety of histopathological features seen in pulmonary carcinoid tumors, it would be reasonable to hypothesize that lung carcinoid tumor is not a pathobiologically homogeneous entity [8]. It has long been known that there are some clinical and pathological differences between carcinoid tumors arising in the bronchus and those located in the periphery of the lung. In this study, we analyzed a variety of clinical, morphological, and immunohistochemical features in a large number of resected lung carcinoid tumors to investigate whether central and peripheral locations could signify biological relevance.

## Materials and Methods

Lung carcinoid tumors were retrieved from histopathology archives during the period from 1994 to 2016. Inclusion criteria for the study were a primary lung carcinoid tumor treated by resection, availability of histologic slides, and follow-up information. A total of 166 tumors were collected and analyzed. The study was approved by the research ethics committee.

Hematoxylin and eosin (H&E)-stained sections from resected tumors were reviewed, and according to the 2015 World Health Organization classification of lung tumors [1], histologic diagnosis of carcinoid tumor was confirmed and sub-classification to typical (TC) and atypical carcinoids (AC) was made. All tumors were evaluated for a variety of histo-morphological features described below.

The tumor was classified as central carcinoid, when the tumor was located in the bronchus (cartilage bearing airway), and classified as peripheral carcinoid when the tumor did not arise from the bronchus and instead was associated with bronchioles. Architectural growth patterns such as organoid nesting, trabecular, insular (lobular), acinar, and papillary were documented in each tumor. Each tumor was divided to circumscribed and infiltrative growth patterns. If there was focal infiltrative growth in otherwise predominantly well-circumscribed tumor, it was regarded as infiltrative. Cell type was divided to polygonal, spindle, and mixed polygonal and spindle types. Non-spindle shapes, including cuboidal, columnar, and plasmacytoid morphologies, were included in polygonal cell type.

Ki67 immunohistochemistry (clone MIB1, 1:50, Dako, Carpinteria, USA) was performed in all tumors. The assessment of mitotic count and Ki-67 index was based on the European Neuroendocrine Tumor Society (ENETS) scheme for gastrointestinal neuroendocrine tumors [9]. Briefly, the mitotic count in 2 mm^2^, that is 10 high power fields (×400) by NIKON Eclipse, was evaluated in areas of the highest mitotic density. Ki-67 indices were calculated as a percentage of Ki-67-positive cells in 500–2000 cells that were counted in areas of the strongest nuclear labeling (“hot spots”). TTF-1 (clone 8G7G3/1, 1:200, Dako, CA, USA) and OTP (polyclonal, HPA039365, 1: 150, Atlas Antibodies, Stockholm, Sweden) stains were also performed according to the manufacturer’s instructions. For both stains, if more than 5% of the tumor expressed a positive reaction in a given tumor, the reaction was regarded as positive, regardless of the staining intensity. Of note, in this study, clone 8G7G3/1 was chosen due to its high specificity, as compared to clone SPT24, which is highly sensitive but less specific.

Presence of sustentacular cells was evaluated in Sox10 staining (clone BC34, 1:200, Biocare Medical, Concord, CA, USA). When more than five Sox10 sustentacular cells were confirmed in each of at least two separate high power fields, the case was regarded as positive for sustentacular cells.

There are no official criteria for neuroendocrine hyperplasia. In this study, as modified from the definition used by Wirtschafter et al. [10], neuroendocrine hyperplasia was diagnosed based upon the presence of at least five neuroendocrine cells in a minimum of three separate airways, which were away from the main tumor and which were present in a tissue block that did not contain the tumor. Neuroendocrine hyperplasia presented as any of the three forms, that is, linear hyperplasia, nodular hyperplasia, and tumorlet. Immunostain for synaptophysin (clone 27G12, 1:50, Novocastra, Newcastle, UK) was reviewed to confirm the presence of neuroendocrine hyperplasia.

Diffuse idiopathic pulmonary neuroendocrine hyperplasia (DIPNECH), which is a generalized proliferation of pulmonary neuroendocrine cells [1], was defined by multiple foci of neuroendocrine hyperplasia along with more than three foci of carcinoid tumorlet [11].

Recorded clinical parameters included tumor size, TNM stage (AJCC 7th edition), and status of surgical margins.

Classification into subgroups was performed by unsupervised hierarchical clustering with between groups linkage as method and squared Euclidean distance as measure. Only histopathological parameters were included in the analysis: histological subtype, Ki-67 index, tumor outline (infiltrative vs. circumscribed), acinar pattern of growth, sustentacular cells, tumor cell type, neuroendocrine hyperplasia, DIPNECH, TTF-1 expression, and OTP expressions. The selection of the best clusters was performed visually based on the dendrogram that was created by hierarchical clustering. An acceptable number of clusters was defined as two to four, considering the number of cases included in the study, and then, the best parameters classifying cases into the clusters were selected in order to search a histological subgrouping.

Comparison between groups was performed by parametric and non-parametric tests. The prognostic significance of the proposed subgrouping as well as baseline demographic, clinical, and histopathological characteristics was examined by Kaplan-Meier curves, Log-rank test, and Cox proportional hazard models. Time to relapse (TTR) was selected as endpoint to assess prognosis, because it does not depend on deaths that might occur from causes other than carcinoid tumor progression. TTR was defined as the time from the surgical tumor resection to the date of relapse or the date of the last follow-up in disease-free status. Patients, who were dead of other causes, were censored at the date of death. Of 159 patients who were disease-free postoperatively, 153 received regular follow-up, with no adjuvant treatment administered. The forward selection procedure with a selection criterion of *p* < 0.10 was used in multivariable Cox proportional hazard models. The parameters examined were age (≥ 60 vs. < 60 years), sex (male vs. female), pT stage (T2–T4 vs. T1), pN stage (N+ vs. N0), tumor location (peripheral vs. central), histology (atypical vs. typical), Ki-67 index (≥ 5% vs. < 5%), tumor contour (infiltrative vs. circumscribed), acinar growth pattern (present vs. absent), sustentacular cells (present vs. absent), cell type (spindle/mixed vs. polygonal), neuroendocrine hyperplasia and DIPNECH (present vs. absent), and TTF-1 and OTP (positive vs. negative). All tests were two-sided with *p* < 0.05 defined as statistically significant. The statistical analysis was performed using Statistical Package for the Social Sciences for Windows version 22 (SPSS, Inc., Chicago, IL).

## Results

### Clinical and Pathologic Features

All patients underwent surgical resection of their primary tumor with curative intent; 93 (56.0%) underwent lobectomy, 21 (12.7%) bilobectomy, 5 (3.0%) tracheobronchial sleeve resection, 5 (3.0%) segmentectomy, 38 (22.9%) wedge resection, and 4 (2.4%) pneumonectomy. One patient underwent two operations for two separate primary tumors in left upper and lower lobes, treated by lobectomies, 42 years apart.

The clinical and pathological characteristics are listed in Table 1. There were 62 males and 104 females (*M*:*F* = 1:1.68). Patient age at initial surgery ranged from 16 to 83 years, with a median age of 59 years.

**Table 1:** Baseline demographic, clinical, and histopathological characteristics

Characteristic	Value	Number	Percent
Age (years)	Median (range)	59	(16–83)
Sex	Male	62	37.3
	Female	104	62.7
pT stage	1	106	63.9
	2	57	34.3
	3	2	1.2
	4	1	0.6
pN stage	0	121	72.9
	1	15	9.0
	2	5	3.0
	NE	25	15.1
Metastatic	Yes	159	95.8
	No	7	4.2
Size (mm)	Median (range)	18	(6–73)
Location	Central	80	48.2
	Peripheral	86	51.8
Histology	Typical	132	79.5
	Atypical	34	20.5
Mitotic rate	Median (range)	0	(0–9)
Ki-67 index	Median (range)	2.0	(0.2–15.0)
Ki-67 index	< 5%	139	83.7
	≥ 5%	27	16.3
Necrosis	Yes	8	4.8
	No	158	95.2
Infiltrative growth	Yes	47	28.3
	No	119	71.7
Acinar growth	Yes	23	13.9
	No	143	86.1
STC	Yes	83	50.0
	No	83	50.0
Cell type	Polygonal	102	61.4
	Spindle	37	22.3
	Mixed	27	16.3
NEH	Yes	37	22.3
	No	115	69.3
	Unknown	14	8.4
DIPNECH	Yes	16	9.6
	No	150	90.4
TTF-1	Positive	82	49.4
	Negative	84	50.6
OTP	Positive	138	83.1
	Negative	28	16.9
Total		166	100.0

*DIPNECH*, diffuse idiopathic neuroendocrine cell hyperplasia; *NE*, Not evaluated; *NEH*, neuroendocrine cell hyperplasia; *STC*, sustentacular cells

**Fig. 1:**
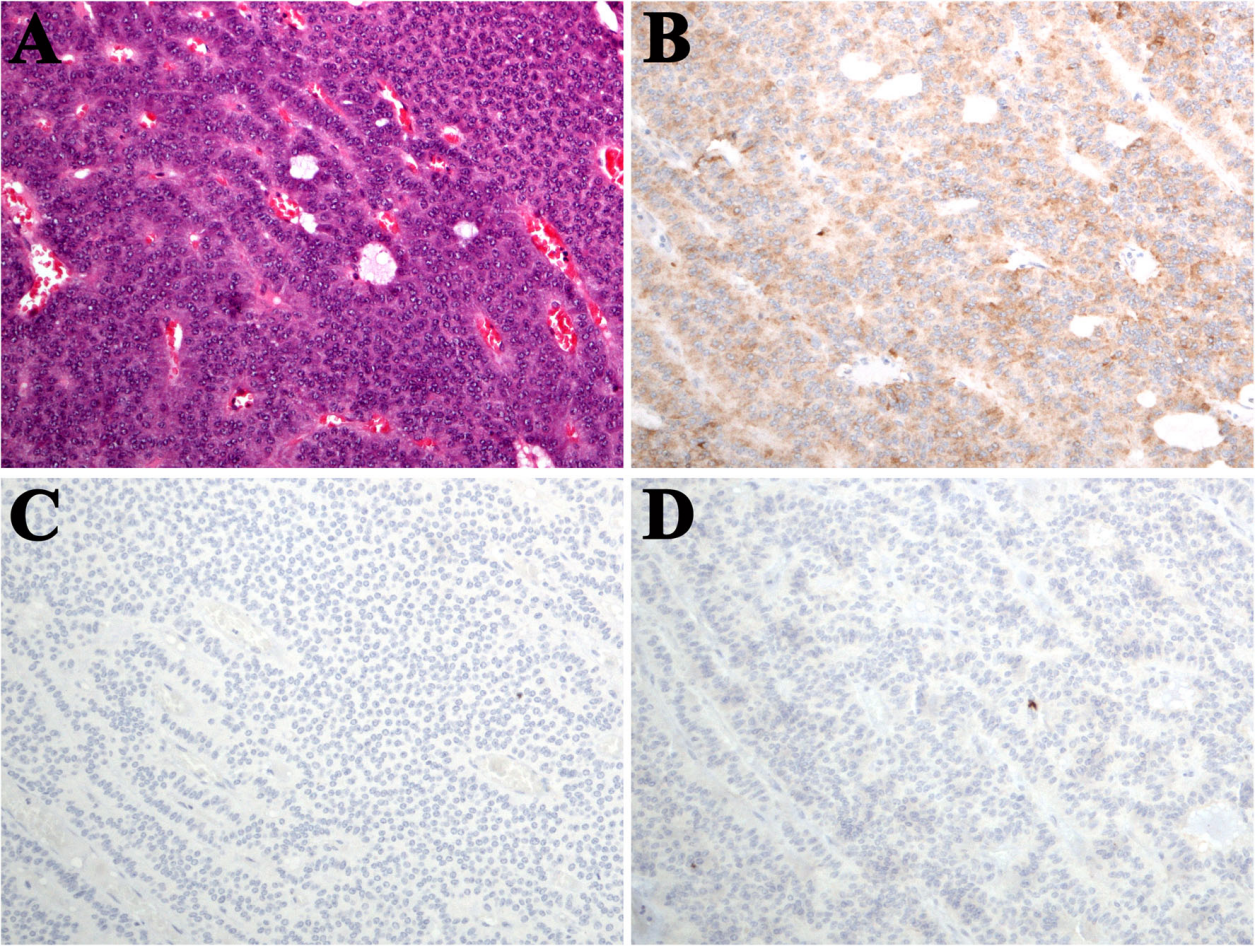
**a** Typical carcinoid in main bronchus, polygonal cell morphology, and closely packed nests and lobules with focal acinar formation (hematoxylin and eosin/H&E stain); **b** OTP negative expression; **c** TTF-1-negative expression; and **d** no sustentacular cells seen in Sox10 staining

**Fig. 2:**
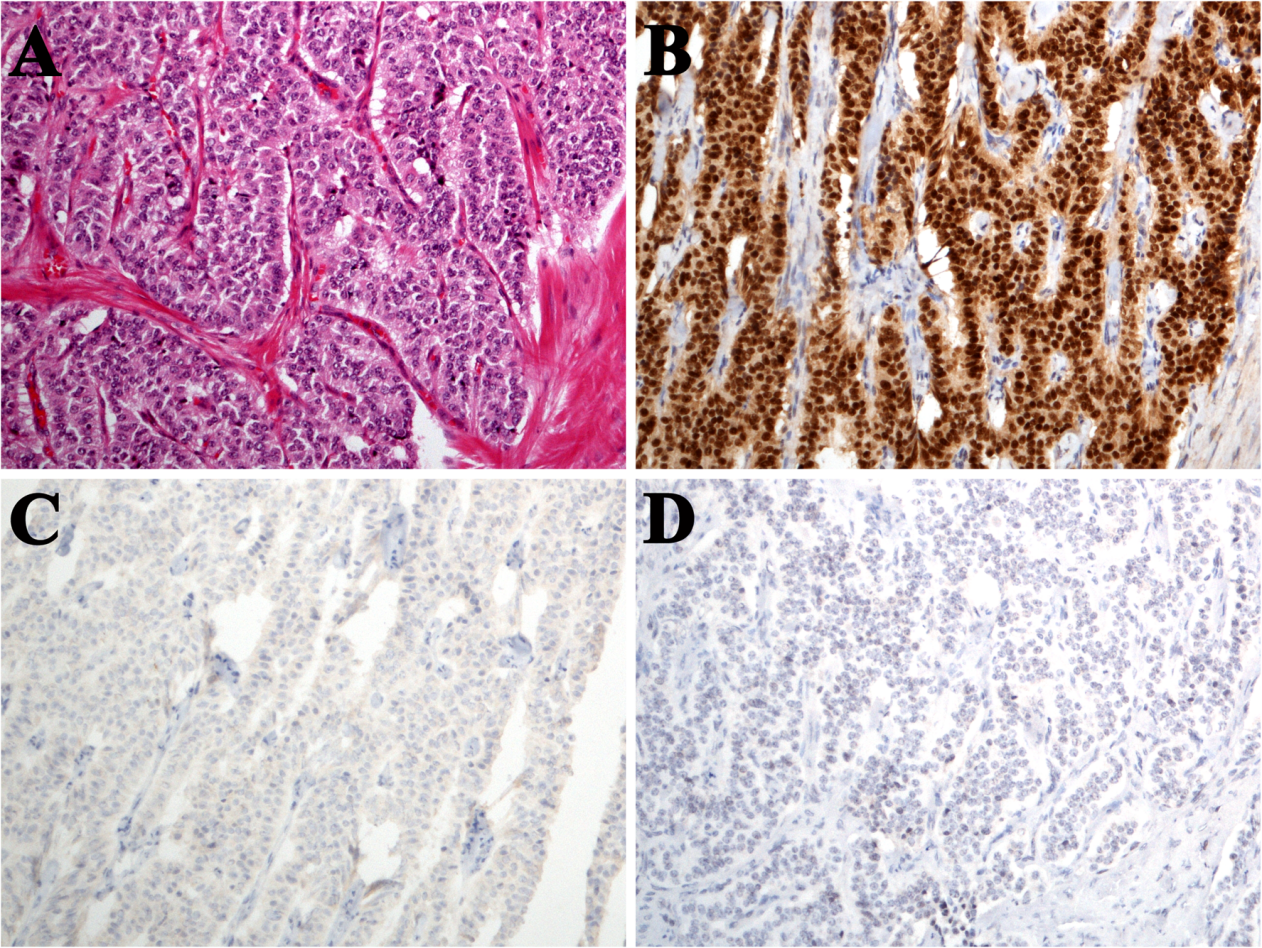
**a** Typical carcinoid in central location, polygonal to cuboidal cell morphology, and trabecular and nesting patterns (H&E); **b** diffuse OTP nuclear staining; **c** no TTF-1 staining, and **d** no sustentacular cells seen in Sox10 staining

**Fig. 3:**
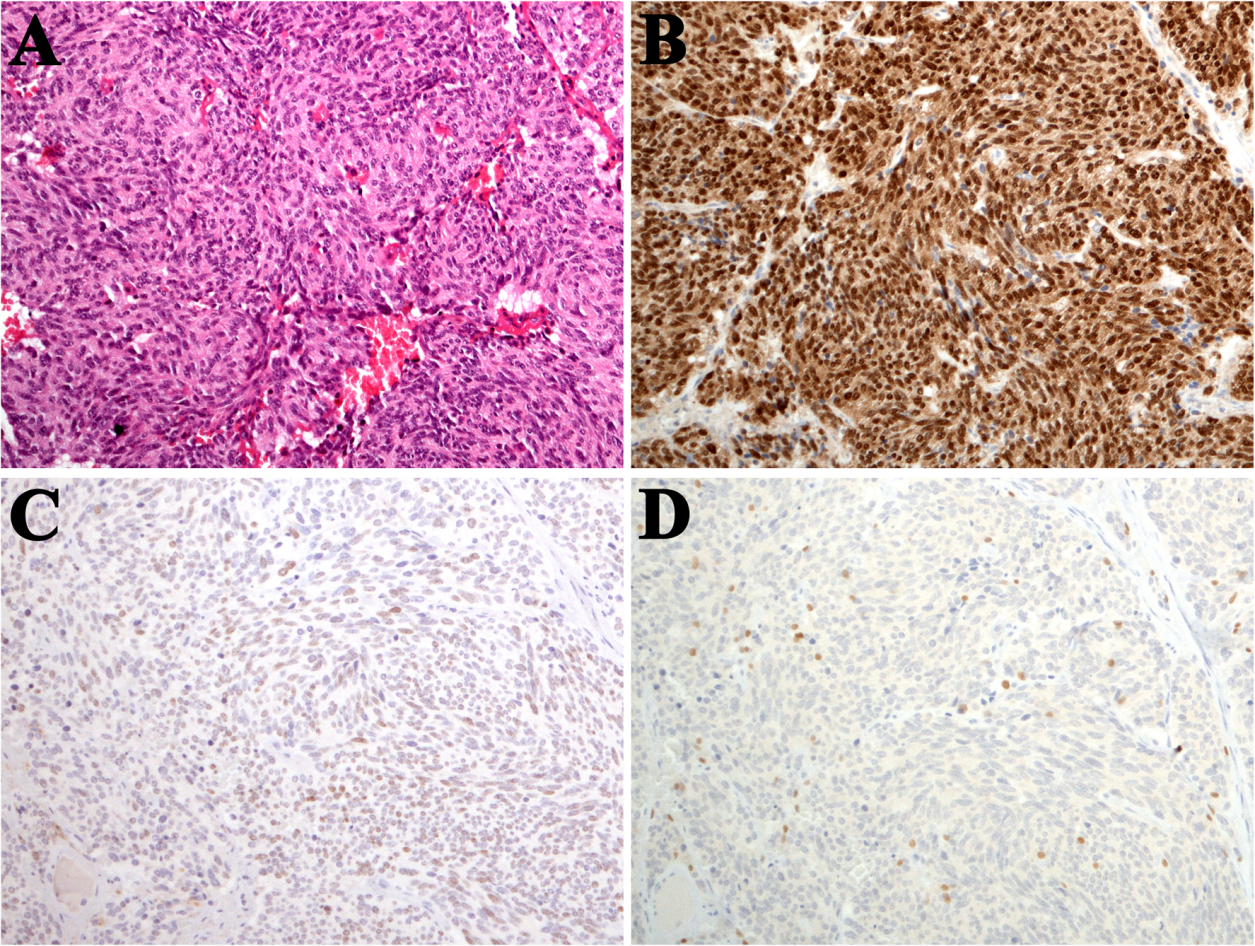
**a** Typical carcinoid in peripheral location, **b** diffuse OTP nuclear staining, **c** focal and weak TTF-1 expression, and **d** scattered Sox10-positive sustentacular cells

The tumor size ranged from 6 to 75 mm with a median of 18 mm. Only one growth pattern was seen in 77 tumors (46.4%). Among them, 75 tumors exclusively showed an organoid nesting pattern while the other two showed only a trabecular pattern. Forty-eight tumors contained two growth patterns, and 41 tumors contained more than 3 patterns. Acinar (glandular) growth pattern, characterized by true luminal structure, was seen in 23 tumors (13.9%) (Fig. 1a–d). Typically, the central tumor, usually, with endobronchial growth, comprised polygonal cells (Figs. 1a–d and 2a–d) while the peripheral tumor contained variable number of spindle cells (Fig. 3a–d). There were 132 typical carcinoid tumors and 34 atypical carcinoid tumors. The Ki67 ranged from 0.2 to 15% with a median of 2%. Sustentacular cells were seen in 83 tumors (50%).

### Statistical Features

#### Subgrouping of Cases According to Basic Characteristics

**Fig. 4:**
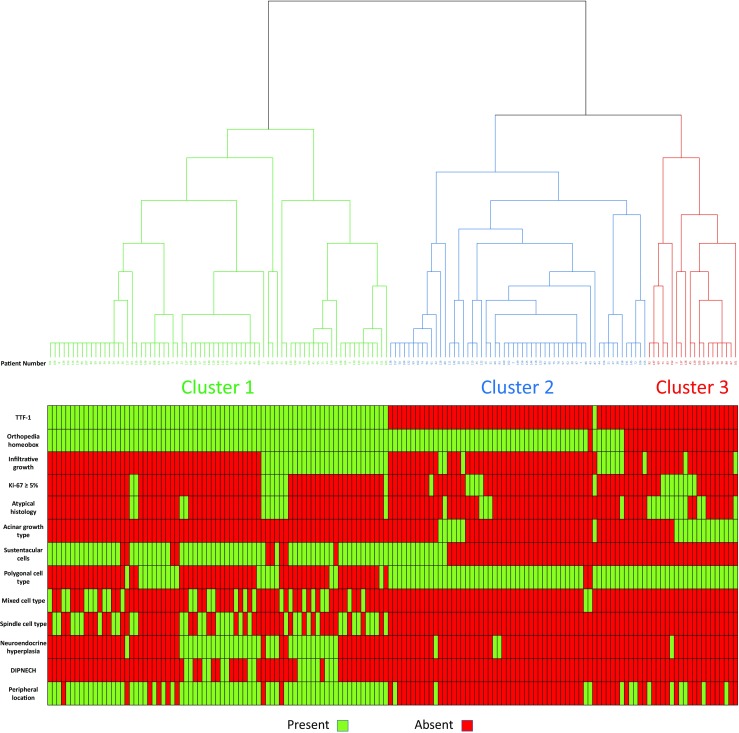
Dendrogram created by unsupervised hierarchical cluster analysis, showing three clusters. The pattern of clinico-pathological characteristics is graphically demonstrated case by case for each cluster

We aimed at subdividing lung carcinoid tumors into groups with distinct characteristics using an unbiased statistic method; hence, unsupervised hierarchical cluster analysis was chosen for this purpose. The method created a dendrogram, where each branch represented a group of similar cases, according to certain characteristics that were used for the clustering, and each group (branch) was further subdivided to subgroups (sub-branches) of more similar cases. The respective dendrogram is demonstrated in Fig. 4. The pattern of clinical and histopathologic characteristics is graphically demonstrated in the same figure.

Hierarchical clustering analysis suggested three clusters as the best solution. The parameters that made the best discrimination between the three clusters were selected based on the pattern of characteristics of each cluster as shown in Fig. 4. First, the presence or absence of TTF-1 expression distinguished almost perfectly cluster 1 from clusters 2 and 3. Importantly, all the TTF-1-positive cases were also positive for OTP (cluster 1). Among TTF-1-negative cases, those allocated in cluster 3 were all OTP negative, while the great majority of cluster 2 was OTP positive. All the other parameters were less accurate in distinguishing between different clusters. Therefore, the above two parameters, namely TTF-1 and OTP, could be proposed as practical tools to classify cases in three subtypes. These subtypes could be named as TTF-1 positive/OTP positive (cluster 1), TTF-1 negative/OTP positive (cluster 2), and finally TTF-1 negative/OTP negative (cluster 3). The pattern of baseline characteristics in the three different lung carcinoid subtypes is described in Table 2. As shown in Table 2, many statistically significant differences in baseline characteristics were identified between these three subtypes. Below, we describe the clinical and histopathological characteristics of the three subtypes.

**Table 2:** Baseline clinical and histopathological characteristics of the three proposed histological subtypes of pulmonary carcinoid tumors

Characteristic	Value		Subtype			*p* value (1)	*p* value (2)	*p* value (3)
			TTF-1(+)/OTP(+)	TTF-1(−)/OTP(+)	TTF-1(−)/OTP(−)			
Age (years)	Median	(Range)	65.5 (22–83)	42 (16–76)	64.5 (37–79)	< 0.001	0.525	< 0.001
Sex	Male	*N* (%)	14 (17.1)	24 (42.9)	24 (85.7)	0.002	< 0.001	< 0.001
	Female	*N* (%)	68 (82.9)	32 (57.1)	4 (14.3)			
pT stage	1	*N* (%)	59 (72.0)	31 (55.4)	16 (57.1)	0.158	0.331	0.775
	2	*N* (%)	21 (25.6)	24 (42.9)	12 (42.9)			
	3	*N* (%)	1 (1.2)	1 (1.8)	0 (0.0)			
	4	*N* (%)	1 (1.2)	0 (0.0)	0 (0.0)			
pN stage	0	*N* (%)	53 (64.6)	45 (80.4)	23 (82.1)	0.205	0.333	0.784
	1	*N* (%)	9 (11.0)	4 (7.1)	2 (7.1)			
	2	*N* (%)	3 (3.7)	2 (3.6)	0 (0.0)			
	NE	*N* (%)	17 (20.7)	5 (8.9)	3 (10.7)			
Metastatic	No	*N* (%)	77 (93.9)	56 (100.0)	26 (92.6)	0.117	0.828	0.108
	Yes	*N* (%)	5 (6.1)	0 (0.0)	2 (7.1)			
Size (mm)	Median	(Range)	18 (6–60)	17 (6–72)	25 (12–75)	0.699	0.007	0.011
Location	Central	*N* (%)	9 (11.0)	52 (92.9)	19 (67.9)	< 0.001	< 0.001	0.008
	Peripheral	*N* (%)	73 (89.0)	4 (7.1)	9 (32.1)			
Histology	Typical	*N* (%)	68 (82.9)	49 (87.5)	15 (53.6)	0.630	0.004	0.001
	Atypical	*N* (%)	14 (17.1)	7 (12.5)	13 (46.4)			
Mitotic rate	Median	(Range)	0 (0–7)	0 (0–6)	1 (0–9)	0.241	0.001	< 0.001
Ki-67 index	Median	(Range)	1.9 (0.2–15.0)	1.8 (0.2–5.8)	3.1 (0.2–10.0)	0.690	0.046	0.019
Ki-67 index	< 5%	*N* (%)	70 (85.4)	50 (89.3)	19 (67.9)	0.611	0.053	0.031
	≥ 5%	*N* (%)	12 (14.6)	6 (10.7)	9 (32.1)			
Necrosis	Yes	*N* (%)	3 (3.7)	1 (1.8)	4 (14.3)	0.646	0.068	0.040
	No	*N* (%)	79 (96.3)	55 (98.2)	24 (85.7)			
Infiltrative growth	Yes	*N* (%)	32 (39.0)	11 (19.6)	4 (14.3)	0.024	0.019	0.764
	No	*N* (%)	50 (61.0)	45 (80.4)	24 (85.7)			
Acinar growth	Yes	*N* (%)	1 (1.2)	6 (10.7)	16 (57.1)	0.018	< 0.001	< 0.001
	No	*N* (%)	81 (98.8)	50 (89.3)	12 (42.9)			
STC	Yes	*N* (%)	69 (84.1)	14 (25.0)	0 (0.0)	< 0.001	< 0.001	0.004
	No	*N* (%)	13 (15.9)	42 (75.0)	28 (100.0)			
Cell type	Polygonal	*N* (%)	20 (24.4.)	55 (98.2)	27 (96.4)	< 0.001	< 0.001	1.000
	Spindle	*N* (%)	37 (45.1)	0 (0.0)	0 (0.0)			
	Mixed	*N* (%)	25 (30.5)	1 (1.8)	1 (3.6)			
NEH	Yes	*N* (%)	33 (40.2)	3 (5.4)	1 (3.6)	< 0.001	0.001	0.804
	No	*N* (%)	43 (52.4)	47 (83.9)	25 (89.3)			
	Unknown	*N* (%)	6 (7.3)	6 (10.7)	2 (7.1)			
DIPNECH	Yes	*N* (%)	16 (19.5)	0 (0.0)	0 (0.0)	< 0.001	0.010	–
	No	*N* (%)	66 (80.5)	56 (100.0)	28 (100.0)			
Total		*N* (%)	82	56	28			
		%	100.0	100.0	100.0			

*p* value (1), TTF-1(+)/OTP(+) vs. TTF-1(−)/OTP(+); *p* value (2), TTF-1(+)/OTP(+) vs. TTF-1(−)/OTP(−); *p* value (3), TTF-1(−)/OTP(+) vs. TTF-1(−)/OTP(−)

*DIPNECH*, diffuse idiopathic neuroendocrine cell hyperplasia; *NE*, Not evaluated; *NEH*, neuroendocrine cell hyperplasia; *STC*, sustentacular cells

##### TTF-1-Positive/OTP-Positive Subtype (Cluster 1)

Tumors in this group were very often located peripherally and contained sustentacular cells. The vast majority of cases were of typical carcinoid subtype and/or had low Ki-67 index. Interestingly, a minority demonstrated infiltrative type of growth, usually not accompanied by atypical carcinoid subtype or high Ki-67 index. Acinar growth was not observed, while approximately three-fourths of the tumors demonstrated pure spindle cell or mixed spindle and polygonal morphology, in other words, characterized by the presence of spindle cell tumor cells in variable proportion. This subtype was much more common in women compared to the other two subtypes, with 82.9% of cases being female. Neuroendocrine hyperplasia was seen in 40% of the cases in this subgroup, and DIPNECH was diagnosed in approximately 20% of the patients, including a patient with known MEN1 (multiple endocrine neoplasia).

##### TTF-1-Negative/OTP-Positive Subtype (Cluster 2)

These tumors were almost always centrally located. The vast majority did not contain spindle cell tumor cells. Indices of biological aggressiveness, such as high Ki-67 index, atypical carcinoid subtype, or infiltrative type of growth, were very unusual in this subtype. A small subset presented acinar growth, while neuroendocrine hyperplasia was scarcely seen and DIPNECH was completely absent. Median age was much younger compared to the other two groups. Male-to-female ratio was 1:1.33.

##### TTF-1-Negative/OTP-Negative Subtype (Cluster 3)

This cluster was the smallest subtype and the most heterogeneous. It included tumors that were located centrally or peripherally. It represented biologically aggressive type, as reflected by a relatively high proportion of tumors with atypical carcinoid histology and/or high Ki-67. Furthermore, acinar growth was more commonly observed than in the other two subtypes, while neuroendocrine hyperplasia was observed in only one case. DIPNECH and sustentacular cells were completely absent, while polygonal cell morphology was seen in the vast majority of cases. Additionally, the median tumor diameter was slightly but statistically significantly larger than in the other two subtypes.

#### Prognostic Significance of Baseline Characteristics and the Histological Subgrouping

**Fig. 5:**
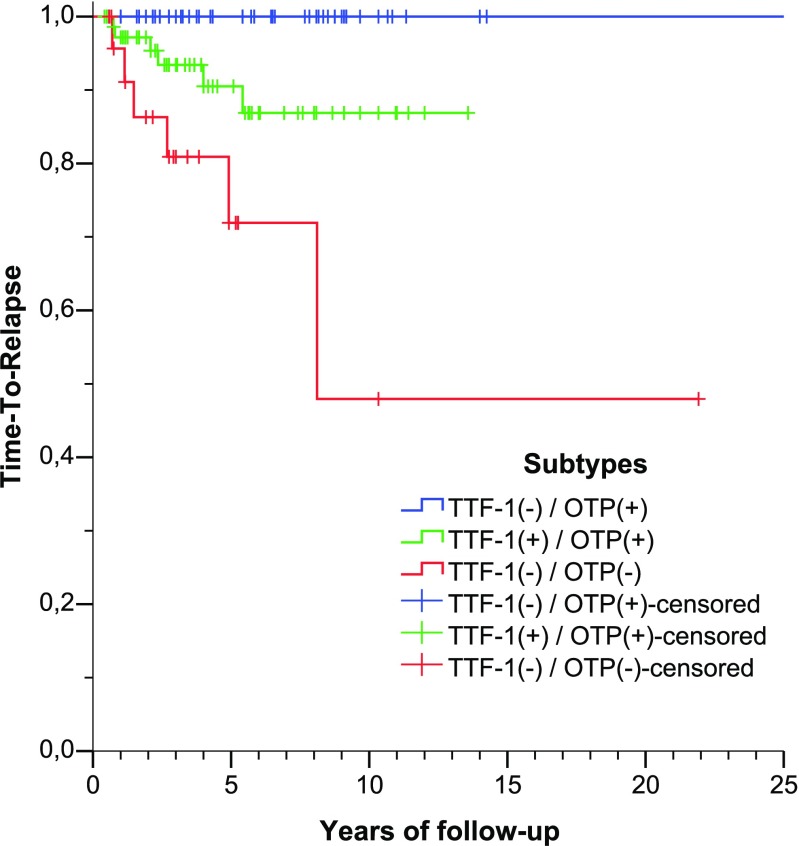
Time-to-relapse curves of the three proposed histological subtypes, defined by the expression of TTF-1 and OTP

Median follow-up was 50 months (range, 5–486 months). Median TTR (time to relapse) was not reached, while estimated 5-year TTR was 91% (95%CI 85–95%). TTR curves were constructed for each proposed subtype. As shown in Fig. 5, TTR curves of different subtypes are clearly separated. TTF-1(−)/OTP(+) subtype had better TTR compared to TTF-1(+)/OTP(+) (chi-square 5.376, *p* = 0.020) and TTF-1(−)/OTP(−) (chi-square 17.473, *p* < 0.001), and TTF-1(+)/OTP(+) subtype had better TTR compared to TTF-1(−)/OTP(−) (chi-square 4.949, *p* = 0.026).

The prognostic impact of the proposed histological subtyping was compared to the prognostic significance of baseline demographic, clinical, and histopathological parameters. In univariable analysis, advanced stage (*p* = 0.007), as well as atypical carcinoid histology (*p* < 0.001), Ki-67 index ≥ 5% (*p* < 0.001), infiltrative growth (*p* = 0.027), acinar pattern (*p* = 0.001), and absence of OTP expression (*p* = 0.001) were unfavorable prognostic factors. Multivariable analysis indicated Ki-67 index ≥ 5% (*p* < 0.001), infiltrative growth (*p* = 0.001), and acinar pattern (*p* = 0.006) as the only independent adverse prognostic factors. As shown in Table 2, the proposed histological subtyping had significantly different frequency of Ki-67 ≥ 5%, infiltrative growth, and acinar pattern, thus explaining their different prognosis.

## Discussion

Neuroendocrine tumors of the lung are a unique group of tumors which share morphological, ultrastructural, and immunohistochemical features, and according to the current WHO classification, classification encompasses four major subtypes, including typical carcinoid (low grade), atypical carcinoid (intermediate grade), large cell neuroendocrine carcinoma (high grade), and small cell carcinoma (high grade) [1]. However, there is growing evidence that carcinoid tumors of both typical and atypical subtypes are pathobiologically distinct from large cell neuroendocrine carcinoma and small cell carcinoma. The current WHO classification is widely accepted by pathologists, surgeons, and oncologists, and histology subtyping by the WHO scheme guides subsequent treatment.

Before the current WHO classification scheme became widely accepted, there were diversity of terminology and inconsistent histopathologic criteria for neuroendocrine tumors of the lung [12]. One of the commonly used schemes subdivided carcinoid tumors to three categories: central, peripheral, and atypical [13] [14]. Indeed, there were a number of case reports and series describing the characteristic features related to the central and peripheral location in the literature. The features often documented in the carcinoid tumors arising in the peripheral location include spindle cell morphology, TTF-1 expression, presence of sustentacular cells, association with neuroendocrine hyperplasia including multiple carcinoid tumorlets or DIPNECH as a clinic-pathologic entity, female gender, and favorable outcome [15–21]. The features often described for the carcinoid arising from the bronchus include rounded (polygonal) shaped cell, TTF-1 negativity, acinar formation, ultrastructural evidence of lumen formation with microvilli, and no significant association with neuroendocrine hyperplasia [7, 15, 22, 23]. With this background in mind, our study was conducted.

Unsupervised hierarchical cluster analysis of our cases led to the three distinct subtypes: (1) TTF-1-positive and OTP-positive type; (2) TTF-1-negative and OTP-positive type; and (3) TTF-1-negative and OTP-negative type. The first type is characterized by female predominance, peripheral location, presence of sustentacular cells, spindle tumor cells in variable proportion, and frequent association with neuroendocrine hyperplasia in the background lung. A subset of the cases was proven to be DIPNECH. This type corresponds to the so-called peripheral carcinoid tumor. The second type, on the other hand, is characterized by central location, polygonal cell morphology, and rare association with neuroendocrine hyperplasia. This subtype corresponds to central carcinoid tumors, many of which often present as an endobronchial tumor. The third type, TTF-1 and OTP double-negative phenotype, was less distinct than the other two types, and a higher proportion of the tumors constituted atypical carcinoid. Indeed, atypical carcinoid constituted 46.4% in this group, as opposed to 17.1 and 12.5% in the first and second groups, respectively. The tumor lacked accompaniment by sustentacular cells and presence of spindle cell component and often comprised polygonal cells and contained acinar growth pattern. The heterogeneous nature of the third group might be attributed to the following backgrounds. It has been shown that loss of OTP nuclear expression is correlated with unfavorable outcome and atypical carcinoid histotype [5, 24, 25]. A subset of the tumors in this group may, indeed, be pathogenetically related to second group (TTF-1-/OTP+) “central carcinoid,” which has lost OTP expression due to transformation to aggressive phenotype. Secondarily, we demonstrated ubiquitous OTP expression in neuroendocrine hyperplasia; however, neither OTP nor TTF-1 (clone 8G7G3/1) was expressed in normal neuroendocrine cells (so-called Kulchitsky cells) of the bronchus [26]. A subset of the tumors in the third group may originate from or differentiate into bronchial neuroendocrine cells.

Parenthetically, given that spindle cell carcinoid and neuroendocrine hyperplasia usually involve the respiratory and terminal bronchioles where a rare structure called neuroepithelial body (NEB) resides [19] and that there is some histo-morphological and ultrastructural similarity between spindle cell carcinoid and neuroendocrine hyperplasia and NEB, the possibility of peripheral carcinoid and tumorlet arising from NEB has been postulated [23, 27], and because of the resemblance between NEB and paraganglia, once the term paraganglioid carcinoid was proposed [21, 22].

There were no cases with TTF-1-positive and OTP-negative phenotype in this series; however, we have experienced two recurrent/metastatic lung carcinoid tumors with TTF-1-positive and OTP-negative phenotype, which were not included in this series. One of them was recurrent atypical carcinoid tumor, with multiple intra-pulmonary metastases. The second tumor was a case of atypical carcinoid in the periphery of the lung, which was completely resected but 8 years later developed multiple metastases in the lung and bone. However, only recurrent tumors were reviewed but the resections of the primary tumor in either case were not available for our review. These cases suggest the existence of TTF-1-positive and OTP-negative phenotype, which might be related to TTF-1/OTP double-positive biologic group with subsequent loss of OTP due to tumor progression.

Interestingly, a few cases of peripheral type carcinoid (TTF-1+/OTP+) occurred in central location, and likewise, central type carcinoid was not always exclusive to the central location; therefore, designation of central and peripheral for such tumors may not be completely appropriate. Review of the literature also reveals uncommon occurrence of pure spindle cell carcinoid arising in the bronchus reported [16, 21, 28].

In summary, the findings in our study confirm that the so-called central and peripheral carcinoid tumors are a subtype of carcinoid tumor with distinct clinical and pathological characteristics. A notable difference is a strong association of the peripheral carcinoid with neuroendocrine hyperplasia, which indicates a different pathogenesis. Histologic features of carcinoid tumor of peripheral type, whether occurring in the peripheral or central location, would require thorough search for underlying neuroendocrine hyperplasia.
